# DLC1 is the principal biologically-relevant down-regulated DLC family member in several cancers

**DOI:** 10.18632/oncotarget.9266

**Published:** 2016-05-10

**Authors:** Dunrui Wang, Xiaolan Qian, Megha Rajaram, Marian E. Durkin, Douglas R. Lowy

**Affiliations:** ^1^ Laboratory of Cellular Oncology, Center for Cancer Research, National Cancer Institute, National Institutes of Health, Bethesda, MD 20892, USA; ^2^ Current address: BioTek Instruments Inc., Winooski, VT 05404, USA

**Keywords:** RhoGAP, tumor suppressor, bioinformatics, DLC genes, TCGA

## Abstract

The RHO family of RAS-related GTPases in tumors may be activated by reduced levels of RHO GTPase accelerating proteins (GAPs). One common mechanism is decreased expression of one or more members of the Deleted in Liver Cancer (DLC) family of Rho-GAPs, which comprises three closely related genes (*DLC1*, *DLC2*, and *DLC3*) that are down-regulated in a wide range of malignancies. Here we have studied their comparative biological activity in cultured cells and used publicly available datasets to examine their mRNA expression patterns in normal and cancer tissues, and to explore their relationship to cancer phenotypes and survival outcomes. In The Cancer Genome Atlas (TCGA) database, *DLC1* expression predominated in normal lung, breast, and liver, but not in colorectum. Conversely, reduced *DLC1* expression predominated in lung squamous cell carcinoma (LSC), lung adenocarcinoma (LAD), breast cancer, and hepatocellular carcinoma (HCC), but not in colorectal cancer. Reduced *DLC1* expression was frequently associated with promoter methylation in LSC and LAD, while *DLC1* copy number loss was frequent in HCC. *DLC1* expression was higher in TCGA LAD patients who remained cancer-free, while low DLC1 had a poorer prognosis than low *DLC2* or low *DLC3* in a more completely annotated database. The poorest prognosis was associated with low expression of both *DLC1* and *DLC2* (P < 0.0001). In cultured cells, the three genes induced a similar reduction of Rho-GTP and cell migration. We conclude that *DLC1* is the predominant family member expressed in several normal tissues, and its expression is preferentially reduced in common cancers at these sites.

## INTRODUCTION

The RHO family of RAS-related GTPases, which includes CDC42, RAC, and RHO, regulates a variety of proliferative, cytoskeletal, and adhesive functions [1], and RHO activity is increased in many advanced cancers [2–3]. Although the RAS GTPases are frequently activated by mutation in tumors [4], such changes are less common among the RHO family GTPases [5–6]. Instead, their high activity is usually attributed to increased function of their activators, the RHO-specific guanine nucleotide exchange factors (GEFs), and/or decreased function of their inactivators, the Rho guanine nucleotide dissociation inhibitors (GDIs) and the RHO-specific GTPase accelerating proteins (GAPs), which hydrolyze the gamma phosphate of active Rho-GTP to inactive Rho-GDP [6]. A few of the 69 RHO-specific GEFs in the human genome have been implicated in a small number of cancers. Among the 64 RHO-specific GAPs, by contrast, reduced expression in cancer has been found frequently among members of the Deleted in Liver Cancer (DLC) family of Rho-GAPs. This family is comprised of three closely related genes: *DLC1* (also known as *ARHGAP7*) [7], *DLC2* (*STARD13*) [8], and *DLC*3 (*STARD8*) [9]. Their encoded Rho-GAP activity strongly hydrolyzes Rho-GTP, weakly hydrolyzes Cdc42-GTP, and has no detectable activity against Rac-GTP [8, 10–11].

Down-regulation of one or more *DLC* genes occurs frequently in a wide range of malignancies. These include solid tumors, such as liver cancer, lung cancer, colorectal cancer, prostate cancer, and breast cancer, as well as several hematopoietic neoplasms [12, 13]. *DLC1* was the first family member identified, and a considerable amount of clinical and experimental evidence has established it as a bona fide tumor suppressor gene. Overexpression of *DLC1* inhibits several biological parameters of neoplastic growth [13], and inactivation of endogenous DLC1 can, in conjunction with other genetic and/or epigenetic changes, lead to cell transformation and tumor formation [14, 15]. *DLC2* and *DLC3* have been studied less extensively, but they also appear to be tumor suppressors that are down-regulated in malignancies [9, 16].

However, it is not known whether the three *DLC* genes are down-regulated with a similar frequency or to the same degree in tumors. Furthermore, in normal cells, it is not clear whether their level of expression is similar or whether one of them may predominate in this regard. This is an important question, as down-regulation of a highly expressed tumor suppressor gene may have greater biological consequences than a similar fold reduction of a less highly expressed related gene, provided the genes have comparable tumor suppressor activities.

To address these issues, here we have taken advantage of The Cancer Genome Atlas (TCGA) database, which includes quantitative RNA-Seq data for expression of the *DLC* genes in a variety of tumors as well as in the respective adjacent normal tissues. In addition, we have experimentally compared the ability of the three DLC proteins to negatively regulate biological and biochemical parameters associated with neoplastic growth. Our analyses indicate that *DLC1* expression is higher than *DLC2* and *DLC3* in several normal tissues, and that, in tumor types arising in these tissues, it is down-regulated to a degree that is greater than or equal to the down-regulation of *DLC2* and *DLC3*. Furthermore, we have found experimentally that the biological activity of the three *DLC* proteins may be similar. These analyses lead us to conclude that down-regulation of *DLC1* often makes a greater contribution to the tumor phenotype than that of *DLC2* or *DLC3*.

## RESULTS

### Down-regulation of *DLC1*, *DLC2*, and *DLC3* in lung, liver, breast, and colorectal cancers

**Figure 1:**
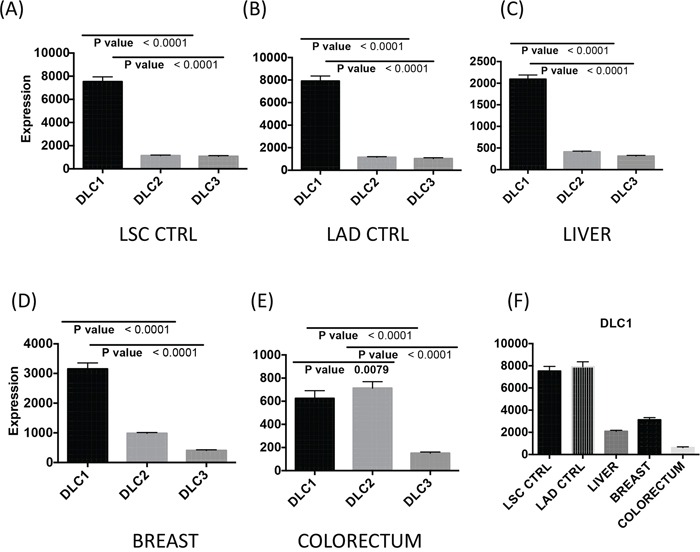
*DLC1*, *DLC2* and *DLC3* gene expression in control tissue adjacent to the tumors Basal RNA expression levels of *DLC1*, *DLC2* and *DLC3* from normal tissue in lung **A**. and **B.**, liver **C.**, breast **D.** and colorectum **E.** are derived from the TCGA dataset (RNA-Seq Version 2, Level 3). The vertical axis differs for some panels. The mean and standard errors of adjacent controls from correspondent cancer have been plotted. **F.** *DLC1* gene expression in different tissues. LSC = lung squamous cell carcinoma; LAD = lung adenocarcinoma.

Using TCGA data from tumor and normal tissue samples of lung squamous cell carcinoma (LSC), lung adenocarcinoma (LAD), hepatocellular carcinoma (HCC), breast adenocarcinoma, and colorectal adenocarcinoma, we first evaluated the RNA expression (RNA-Seq Version 2) of the three *DLC* family members in the normal tissues adjacent to the tumors. In each of the normal tissues except the colorectum, *DLC1* was more highly expressed than *DLC2* and *DLC3* (Figure 1A–1E). *DLC1* expression was 7 times higher than *DLC2* and *DLC3* in lung, 3 times higher in breast, and 4 times higher in liver. Reassuringly, the respective expression of all three *DLC* genes was similar in the normal lung tissues from the two forms of lung cancer LSC and LAD (Figure 1A and 1B). *DLC1* expression in the lung was substantially higher than in the liver and breast (Figure 1F). In the normal colorectum, *DLC1* expression was substantially lower than in the other tissues (Figure 1F), but it was still about 3-fold higher than *DLC3* (Figure 1E). However, the levels of *DLC1* and *DLC2* were similar.

**Figure 2:**
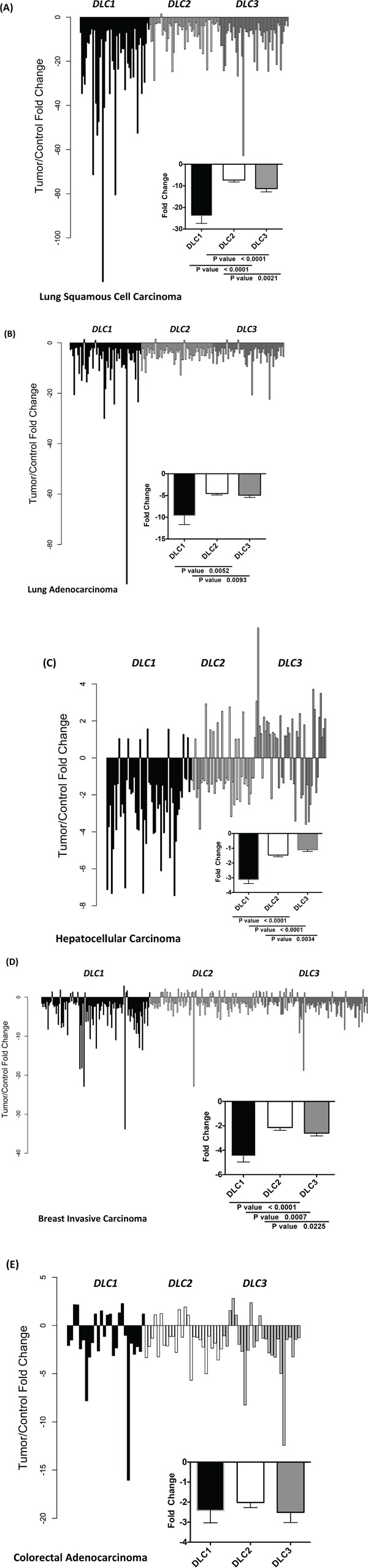
Fold change in *DLC1*, *DLC2*, and *DLC3* expression between tumor and adjacent control tissue The fold change of *DLC1*, *DLC2* and *DLC3* RNA-Seq Version 2 values from individual paired control to tumor of the TCGA dataset are plotted for lung squamous cell carcinoma **A.**, lung adenocarcinoma **B.**, hepatocellular carcinoma **C.**, breast cancer **D.** and colorectal adenocarcinoma **E.**

We then compared the changes in expression between paired cancer and adjacent normal samples (Figure 2). In LSC and LAD, *DLC1* expression was reduced 24-fold and 10-fold, respectively, while the fold reduction for *DLC2* and *DLC3* in both of these tumor types was less than one-half as much (Figure 2A and 2B). The magnitude of these reductions in *DLC1* expression was striking, especially as its expression level in the normal tissues was highest in lung. In order to determine the percentage of patients with low *DLC* expression for LAD and LSC, we compared *DLC* values from LAD and LSC to the cutoffs determined by lung controls. We found that 46% of LAD and 96% LSC had low *DLC1*, 86% of LAD and 95% of LSC had low *DLC2* and 72% of LSC had low *DLC3* if cutoffs were defined by mean minus 2 standard deviations. One hundred percent of both LAD and LSC had low *DLCs* if cutoffs were defined by mean minus 1 standard deviation. In HCC, there was a 3-fold reduction in *DLC1* expression, while *DLC2* and *DLC3* expression was not reduced, in part because there was increased expression of *DLC2* and, especially, *DLC3* in a substantial number of the tumors (Figure 2C). In breast cancer, the reductions were 4-fold for *DLC1* and 2-fold for both *DLC2* and *DLC3* (Figure 2D). Analysis of breast cancer subtypes indicated that the expression of *DLC1*, *DLC2*, and *DLC3* was significantly lower in triple-negative breast cancer (TNBC) than in the other subtypes (Figure S1). The reduction in *DLC2* expression in triple-negative breast cancer was greater than that of *DLC1* or *DLC3*. In colon cancer, where *DLC1* expression had not predominated in the normal tissue, there was a 2-fold reduction for all three *DLCs* genes (Figure 2E).

**Figure 3:**
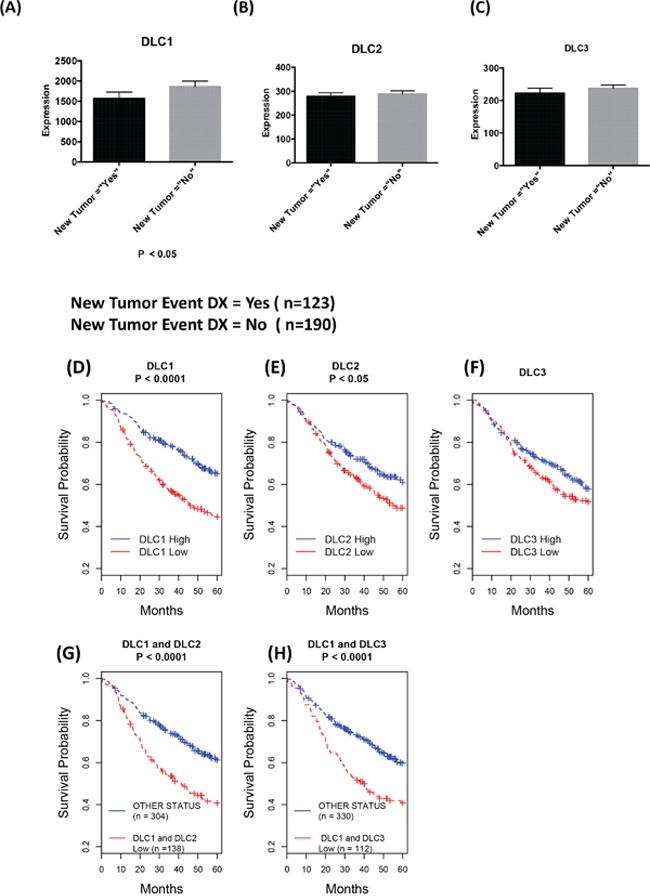
Down-regulation of *DLCs* is associated with poor prognosis **A-C.** Comparison of DLC gene expression of patients with follow up status based on “new tumor event dx indicator” of TCGA lung adenocarcinoma clinical data as of October 2015. The most recent clinical patient status has been selected, and *DLC* gene expression (RNA-Seq Version 2) mean and standard errors of the mean are plotted against “new tumor” status. **D-H.** Kaplan-Meier survival analysis: Down-regulation of *DLC1* and *DLC2* is associated with poor prognosis. From the Director's Challenge Lung Study cohort of 442 lung adenocarcinomas. High and Low in the Figure legend represent the status of the mRNA expression level compared to the median of the expression for corresponding gene. **D-F.** Survival comparison between patients with low vs. high expression of the designated *DLC* gene. **G.** Survival comparison between patients whose *DLC1* and *DLC2* expressions are low vs. all others. **H.** Survival comparison between patients whose DLC1 and DLC3 expressions are low vs. all others.

In the TCGA dataset, clinical follow-up divided patients between those who did, and those who did not, develop a new tumor. In LAD, *DLC1* expression, but not *DLC2* and *DLC3* expression, was higher in the “No New Tumor” group than in the “New Tumor” group (Figure 3A-3C). No such differences in *DLC* gene expression were observed in LSC and breast cancer (data not shown).

To evaluate in more detail the possible relationship in LAD between prognosis and expression of the three *DLC* genes, we took advantage of the Director's Challenge cohort caArray dataset of 442 LAD cases (jacob-00182) [17], whose annotated survival information is more extensive than the TCGA dataset. We have previously used this dataset to determine that low *DLC1* expression is associated with a poor prognosis [22]. In addition to confirming this result (Figure 3D), we found that low *DLC2* expression (Figure 3E) was also associated with an adverse outcome, although not to the same degree as *DLC1* (P = 3E-06 for *DLC1* vs. P = 0.015 for *DLC2*). Low DLC3 expression was not associated with clinical outcome (P = 0.20 for *DLC3*, Figure 3F). The combination of low *DLC1* and low *DLC2* (Figure 3G) or low *DLC1* and low *DLC3* (Figure 3H) was not a better predictor of outcome than that of low *DLC1* by itself (P = 8E-06 for *DLC1/DLC2* and P = 7E-05 for *DLC1/DLC3*).

### Down-regulation of *DLCs* is associated with copy number loss and promoter methylation

**Figure 4:**
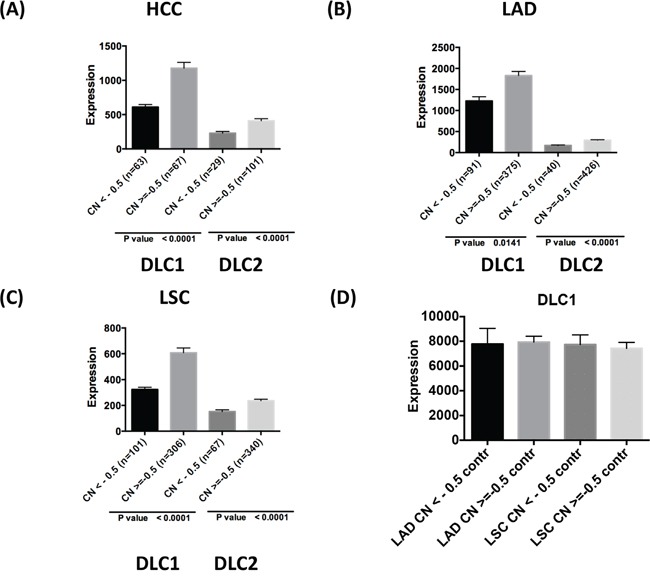
*DLC* copy number variation and gene expression in tumors Comparison of *DLC1* and *DLC2* RNA-Seq values and copy number (CN) variation in TCGA HCC **A.**, LAD **B.**, and LSC **C.** The patients are grouped based on copy number loss variation (value log2 <-0.5 and log2 > = −0.5). **D.** *DLC1* expression in adjacent control lung tissue grouped according to the copy number status of the respective LAD and LSC tumors.

*DLC1* maps to the 8p21.3-22 chromosome region, which is frequently deleted in a number of human tumor types [7, 23]. In addition to deletion of *DLC1*, reduced expression of *DLC1* in cancer has also been linked to promoter hypermethylation [12–13]. However, the relative contribution of these genetic and epigenetic changes to DLC expression has not been examined. In the TCGA HCC dataset, close to one-half (48%) of the tumors had *DLC1* copy number loss, while just under one-quarter (22%) of them had copy number loss for *DLC2* (Figure 4A). Expression of *DLC1* and *DLC2* was two-fold lower in the groups with copy number loss than in those without it. Compared with HCC, copy number loss of *DLC1* was less frequent in LAD (20%) and LSC (25%) (Figure 4B and 4C), and, as expected, was not present in control tissue (Figure 4D). *DLC2* copy number loss was also less frequent for LAD (9%) and LSC (16%). As with HCC, down-regulation of *DLC1* and *DLC2* expression was found in the groups with CNV log2 < −0.5 in LAD and LSC. No *DLC3* copy number loss was seen in HCC, LAD, or LSC (data not shown).

**Figure 5:**
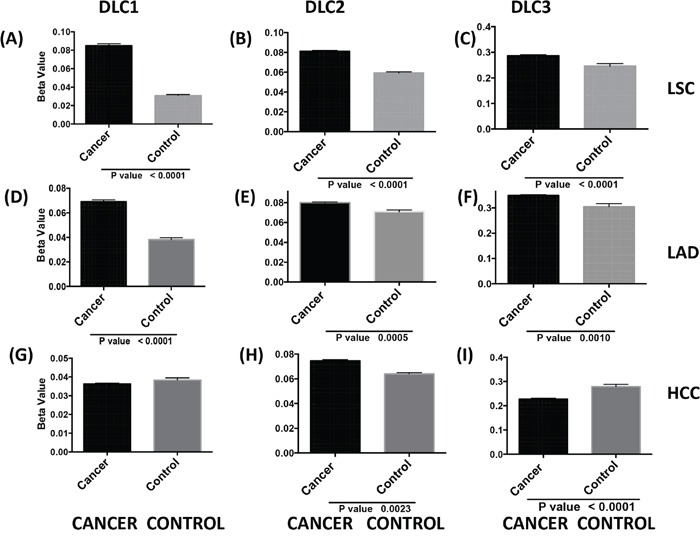
*DLC* promoter methylation and gene expression in tumors TCGA level 3 data from JHU_USC__ HumanMethylation450 directory of each selected cancer were used for analysis. *DLC* means and standard errors of cancer and controls in LSC **A-C.**, LAD **D-F.** and HCC **G-I.** were calculated using beta values from all available probes in the *DLC1* variant 2 **A., D., G.** *DLC2* alpha, variant 1 **B., E., H.** and *DLC3* beta variant 3 **C., F., I.** (sequence details in Figure S2).

Each of the three DLC family genes has a predominant transcript (9, 12, 24–25). Hypermethylation of the CpG-rich promoter region of the *DLC1* variant 2 transcript, which is both the most abundant one and the one that has been studied in greatest detail, has been found in a number of cancers [12–13], but less is known about methylation of the *DLC2* and *DLC3* promoters (*DLC* promoter sequences are shown in Figure S2). In the TCGA dataset, the level of *DLC1* methylation was about two-fold higher in LSC and LAD than in normal lung (Figure 5A and 5D). By contrast, there was no difference in the average level of *DLC1* methylation between HCC and normal liver (Figure 5G). The level of *DLC2* and *DLC3* methylation in LSC was significantly higher in tumors than in normal tissue (Figure 5B and 5C). Methylation of individual CpG sites in *DLC1* showed the highest levels in LSC cases, followed by LAD and HCC (Figure 5 and Figure S3). The increased *DLC1* methylation was associated with lower gene expression in LSC and LAD, but not in HCC (Figure S4). For *DLC2*, methylation was increased in LSC (Figure 5B), but less than for *DLC1* (Figure 5A). There were only modest (less than two-fold) differences in *DLC2* and *DLC3* methylation between the tumors and the respective normal tissue, although the increase in methylation was significant for *DLC2* and for *DLC3* in LSC and LAD (Figure 5B, 5C, 5E and 5F). There was actually a small, but significant, decrease in *DLC3* methylation in HCC (Figure 5I). Thus, promoter methylation may make a larger contribution to low *DLC1* expression in LSC and LAD than in HCC, while low *DLC1* expression in HCC is more closely linked to copy number loss.

### *TP53* mutation is associated with low *DLC1* expression in cancer

**Figure 6:**
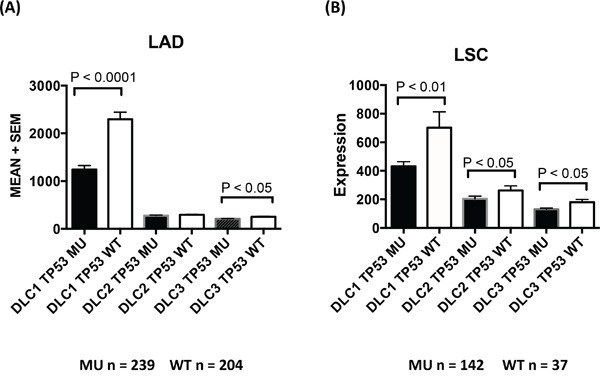
*TP53* mutation and DLC expression in lung adenocarcinoma and lung squamous cell carcinoma TCGA LAD **A.** and LSC **B.** datasets. *DLC* expression levels (mean + standard error) are plotted against groups of patients with or without TP53 mutations. MU = TP53 mutation. WT = TP53 wild type.

We evaluated whether altered expression of any of the three *DLC* genes in the cancers might be associated with mutations in *TP53*, one of the most commonly mutated genes in cancer [26]. Among 520 and 178 TCGA patients with LAD and LSC, there were 276 (53%) and 141 cases (79%), respectively, with at least one *TP53* mutation (including frame shift deletions, frame shift insertions, in-frame deletions, missense mutations, nonsense mutations, and splice site mutations). *DLC1* expression was lower in patients with *TP53* mutations than in patients with wild *TP53* (Figure 6) in LAD and LSC. Expression of *DLC2* and *DLC3* was also lower in patients with mutant TP53, although not to the same degree as with *DLC1*.

Further analysis of the TCGA dataset showed that *DLC1* down-regulation and *TP53* mutation were found more frequently in current smokers with LAD. Fifty-five percent of current smokers had low *DLC1* expression and *TP53* mutations, while only 29% of former smokers and 20% never smokers had this phenotype. The combination of low *DLC1* and *TP53* mutation in current smokers was more frequent than that of low *DLC2* or *DLC3* and TP53 mutation (Figure S5A). Although the Director's Challenge Lung Study cohort dataset does not have information about p53 status, the smoking history of the patients is known. Consistent with the TCGA dataset, analysis of this cohort showed that the average expression among non-smokers (never smokers and former smokers combined) was 1.6-fold higher for *DLC1* compared to current smokers (P < 0.01) (Figure S5B). There was no significant difference for expression of any *DLC* gene between never-smokers and former smokers.

### *DLC1*, *DLC2*, and *DLC3* have comparable biological activities

**Figure 7:**
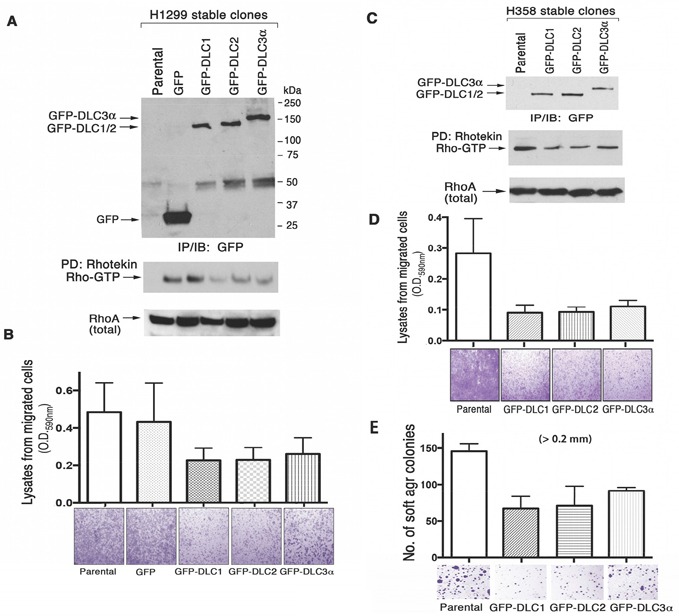
DLC RhoGAP and bioactivity in transfected human lung cancer cell lines GFP-tagged *DLC1*, *DLC2* and *DLC3α* constructs have been stably transfected into H1299 **A.** and **B.** and H358 **C.**, **D.** and **E.** cells. The expression of GFP or GFP-DLCs in the established stable clones has been analyzed by IP followed by IB with anti-GFP antibody. RhoGAP activity was measured by Rhotekin pull-down assay (A and C). In the cell migration assay, the migrated H1299 and H358 transfectants in the botom chamber of 24 well inserts were stained and photographed using a light microscope. The quantitation was performed colorometrically as described in materials and methods (B and D). Equal numbers of H358 stably transfected cells were seeded in soft agar for growth and quantitation as shown to compare the effect of anchorage-independent cell growth (E).

To determine whether the three *DLC* genes have comparable biological activity, we analyzed two LAD cell lines (H1299 and H358) that had been stably transfected with constructs expressing DLC1, DLC2, and DLC3. Each *DLC* gene encoded the same epitope tag (GFP), to be able to verify that the transfectants expressed similar levels of the respective DLC protein. The level of RhoA-GTP was reduced to a similar degree in cells expressing DLC1, DLC2, or DLC3, when compared to cells transfected with vector only (Figure 7A and 7C). In addition, compared to the control cells, the migration rate of the cells expressing the each *DLC* gene was slowed to a similar degree (Figure 7B and 7D), as was the anchorage-independent cell growth in soft agar (Figure 7E). The results suggest that these biochemical and biological activities are comparable between the three *DLC* genes when expressed in cultured cells.

## DISCUSSION

We have used detailed information from TCGA and another cancer database, together with experimental analysis, to evaluate the relative role of the three *DLC* genes in five tumor types: LSC, LAD, breast cancer, HCC, and colorectal cancer. This assessment, which has been made possible by the TCGA database, has led to several main observations. First, *DLC1* is more highly expressed than *DLC2* and *DLC3* in most of the normal tissues examined. Second, except for colorectal cancer, *DLC1* is down-regulated to a greater degree in the tumors, in terms of both the magnitude of the reduction and the fold-reduction compared with *DLC1* expression in the respective normal tissue, than the other two family members. Third, the reduced expression of *DLC1* in LSC and LAD was frequently associated with promoter methylation, while this reduction was frequently associated with *DLC1* copy number loss in HCC. Fourth, under normal growth conditions, the three *DLC* genes negatively regulate Rho-GTP levels and inhibit cell migration and anchorage-independent growth to a similar degree. These observations strongly suggest that the reduced *DLC1* levels in tumors have a greater impact on the biological properties of the tumor types examined than do those of *DLC2* or *DLC3*.

The three *DLC* genes share a similar genomic organization and are closely related evolutionarily. The DLC1 and DLC2 amino acid sequences are 58% identical, and are, respectively, 44% and 52 % identical to DLC3 (9, 12). Previous reports have suggested that *DLC1* is the most widely expressed of the three *DLC* genes, followed by *DLC2*, and *DLC3*, which has a more limited tissue distribution [9, 27]. Here, our analysis found that all three genes are expressed in the four normal tissues examined: lung, breast, liver, and colorectum. The level of *DLC1* expression varied considerably between these tissues; it was more than 10 times higher in normal lung than in normal colorectum. Despite this wide range, it was higher than *DLC2* in each of the tissues except for colorectum, where expression of *DLC1* and *DLC2* was similar, and it was substantially higher than *DLC3* in all four tissues. Taken together, these observations suggest *DLC1* may be the most critical of the three genes for normal physiology, which is consistent with its requirement for fetal development [28–29], in contrast to *DLC2* [30] and *DLC3* [31]. However, *DLC2* and *DLC3* may have cell type-specific functions, such as the role of *DLC2* in pancreatic physiology [32].

When the tumors were analyzed for their expression of the three *DLC* genes, the most striking results were seen in the lung. The mean expression of *DLC1* in LSC and LAD was reduced about 24–fold and 10-fold, respectively, compared with its expression in the normal lung. Although *DLC2* and *DLC3* expression was also reduced in both forms of lung cancer, the fold-reduction of *DLC1* in each tumor type was more than twice that of *DLC2* or *DLC3*. Because the expression of *DLC1* in the non-tumor tissue was so much higher than that of *DLC2* and *DLC3*, the magnitude of the reduction in the number of *DLC1* RNA molecules in the tumors was far larger than that of the reduction in the number of *DLC2* and *DLC3* RNA molecules.

Given our experimental *in vitro* findings that the biological activities of *DLC1*, *DLC2*, and *DLC3* appear to be similar, we conclude that the biology of changes in *DLC1* expression are more relevant to the lung tumors than are those in the other two family members. This conclusion is further supported by prognostic data from an LAD cohort, whose patient outcomes are more completely annotated than those of the TCGA tumors. In that cohort, the patients whose *DLC1* expression was below the median had a poorer prognosis than those whose *DLC2* or *DLC3* expression was below the median for those genes. However, reduced expression of *DLC2*, but not *DLC3*, was associated with a poorer outcome, although not to the same degree as reduced *DLC1* expression.

Reduced expression of the *DLC* genes in cancer has been attributed to several mechanisms. They include gene deletion [8, 12–13, 33], increased promoter methylation [12–13], histone deacetylation [34], histone methylation [35], and decreased protein stability [36]. Prior to the molecular identification of *DLC1*, cytogenetic analysis had indicated that chromosome 8p22, where *DLC1* is located, was frequently deleted in HCC [37]. Our results indicate that copy number loss of *DLC1* occurred about twice as frequently in HCC (48%) as in LSC (25%) or LAD (20%). Compared with *DLC1*, copy number loss of *DLC2* was less common in HCC (22%), LSC (16%) and LAD (9%). *DLC1* and *DLC2* copy number loss percentages in the TCGA breast carcinoma dataset were 33% and 18% respectively (data not shown). No *DLC3* copy number losses were found in any of the tumors examined in this study, although losses at the *DLC3/STARD8* locus at Xq13 have been reported in ovarian carcinomas [38]. As expected, copy number loss of *DLC1* was found to be associated with its reduced expression. Since almost one-half of the HCC cases had *DLC1* copy number changes, this mechanism may be a major factor driving the reduced expression of *DLC1* in this tumor type.

The less frequent *DLC1* copy number losses in LSC and LAD suggest that other mechanisms contribute to the decreased *DLC1* expression in these tumors. Consistent with this possibility, in LSC, the level of CpG methylation at the *DLC1* promoter was increased, and this increase was associated with reduced *DLC1* expression. *DLC1* methylation was also increased in LAD, but to a lesser extent than in LSC. No increase in *DLC1* methylation was observed in HCC. Thus, HCC tumors are more likely to present with *DLC1* copy number loss than with promoter hypermethylation, while the situation is reversed in LSC and LAD. The differences in methylation of the promoter regions of *DLC2* and *DLC3* were less substantial than those observed with the DLC1 promoter. In addition, data from Yuan at al. [39] have shown that the *DLC1* promoter is methylated in some of the NSCLC cell lines that do not express *DLC1* mRNA, and can be re-activated by treatment with azacytidine.

Our results show an association of low *DLC1* levels with *TP53* mutations in the lung tumors, and the combination of low *DLC1* expression and *TP53* mutation was more prevalent in current smokers. An association between *TP53* mutations and expression of the other DLC family members is not striking. Mutations in *TP53* are common in smoking-related cancers [40] and occur more frequently in lung tumors from smokers [41]. Inactivation or mutation of *TP53* is reported to lead to increased Rho GTPase signaling and to the acquisition of a more aggressive, invasive phenotype in tumor cells [42]. Further analysis of the links between *TP53* and *DLC1* expression in cancer may help to elucidate how dysregulation of the p53 and RhoA pathways might cooperate to promote oncogenesis.

We conclude that in several cancers *DLC1* is the principal biologically-relevant down-regulated DLC family member, although down-regulation of *DLC2* and *DLC3* is also observed. This conclusion, which parallels the physiologic observation that *DLC1* is essential for fetal development, while *DLC2* and *DLC3* are dispensable, implies that analyses of the *DLC* genes in cancer analyses should focus preferentially on *DLC1*.

## MATERIALS AND METHODS

### Bioinformatics analysis

The analyses reported in this study employed data from The Cancer Genome Atlas (TCGA, http://cancergenome.nih.gov) and caArray (https://array.nci.nih.gov/caarray/home.action) of National Cancer Institute (NCI), both of which are publicly available. RNA expression (RNA-Seq Version 2), somatic mutation and clinical data (October 2015 release) were downloaded directly from TCGA portal. A cohort from caArray that contains lung adenocarcinomas (jacob-00182) [17] was downloaded, and the CEL files with raw data were normalized using 3′ Expression Arrays Robust Multi-array Analysis (RMA) from the Affymetrix software Expression Console (http://www.affymetrix.com). The normalized expression values represent the probe set intensity on a log-2 scale.

Gene expression comparison (level in normal tissue vs. tumor tissue fold change), Mann-Whitney U test, Kaplan-Meier survival analysis, and Chi Square test (chisq.test) were carried out using the open source statistical tool R (version 2.14.1), Prism program (version 6.0e, GraphPad Software, Inc.), or Microsoft Excel. P value < 0.05 is considered as statistically significant.

For survival analysis and smoking-related analysis, values higher or lower than the median in each gene group were categorized, respectively, as “high” or “low.” All survival times were adjusted to months.

TCGA *DLC* copy number variation (CNV) data (log2 value) were derived from The cBio Cancer Genomics Portal (cBioPortal) [18–19]. The value < −0.5 was designated as copy number loss.

*DLC* methylation data were directly downloaded from TCGA DNA methylation level 3 data sets. TCGA uses results generated with the Illumina HumanMethylation450 BeadChip to report the methylation status of the promoter regions of the *DLC1* variant 2, *DLC2* alpha, variant 1, and *DLC3* beta, variant 3 transcripts, which are analogous to the variant 2 transcript of *DLC1*. When the average beta values (estimate of methylation level using ratio of intensities between methylated and unmethylated alleles) of all detected methylation sites were calculated, the *DLC* promoter regions (9, 9, and 10 sites for *DLC1*, *DLC2*, and *DLC3*, respectively) in LSC, LAD, and HCC and their respective controls were compared.

### Transfection and RhoA activation assay

The detailed methods for transfection and the RhoA activation assay were described previously [20]. Briefly, H1299 and H358 cells were stably transfected with vectors that express green fluorescent protein (GFP)-tagged human *DLC1*, *DLC2* (gift from Dr. Michael Mowat, Manitoba Institute of Cell Biology, Canada), or *DLC3* [20], as well as pEGFP (Clontech Laboratories, Inc.). Cell extracts were collected using magnesium lysis buffer (EMD Millipore) supplemented with 1 mM Na_3_VO4 and protease inhibitor mixture tablet (Roche Diagnostics). Equal amounts of protein lysates were used for pull-down by Rhotekin RBD agarose (EMD Millipore). The pellets were washed three times with lysis buffer, resuspended in Laemmli sample buffer, and then separated by 15% SDS/PAGE. Anti-RhoA antibody (EMD Millipore) was used for immunobloting to detect RhoGTP, and anti-GFP antibody was used to detect GFP-tagged *DLCs* and the GFP control. For each blot, horseradish peroxidase-conjugated anti-rabbit or anti-mouse immunoglobulin A/G (GE Healthcare) was used for the second reaction at 1:10,000 dilution. Immunocomplexes were visualized by enhanced chemiluminescence (ECL), using an ECL kit (GE Healthcare).

### Cell migration assay and soft agar colony growth assay

The cell migration assay was performed using transwell inserts as described previously [21]. Briefly, equal numbers (4×10^4^) of transfected H1299 cells expressing GFP or GFP-tagged DLC1, DLC2 and DLC3 were added to the upper chamber of a 24-well plate. The cells were incubated at 37C° and permitted to migrate to the lower chamber for 16 hours. The cells on the lower surface of the filter were fixed with methanol, stained with 0.5% crystal violet, and examined in a dissecting microscope at high (20X) and low (4X) power. Each experiment was repeated three times. For quantitation, stained membranes were incubated in 1% Triton-X100 solution, and the optical density was measured with a spectrophotometer at 590nm.

For soft agar colony assays, 1 × 10^5^ cells were mixed with complete medium containing 0.4% agar (Difco) and placed over 0.6% basal agar in 60-mm dishes. Cells were grown for 3 weeks, and colonies were photographed microscopically and quantified with a colony counter after staining with 1 mg/ml Nitrotetrazolium Blue Chloride (Sigma-aldrich).

## SUPPLEMENTARY FIGURES


