# Supplementary material for: DLC1 is the principal biologically-relevant down-regulated DLC family member in several cancers
Source: Oncotarget. 2016 May 10;7(29):45144–57. doi: 10.18632/oncotarget.9266 (PMC5216712; doi:10.18632/oncotarget.9266)
Supplement: Supplementary file 1 [file oncotarget-07-45144-s001.pdf]

SUPPLEMENTARY FIGURES

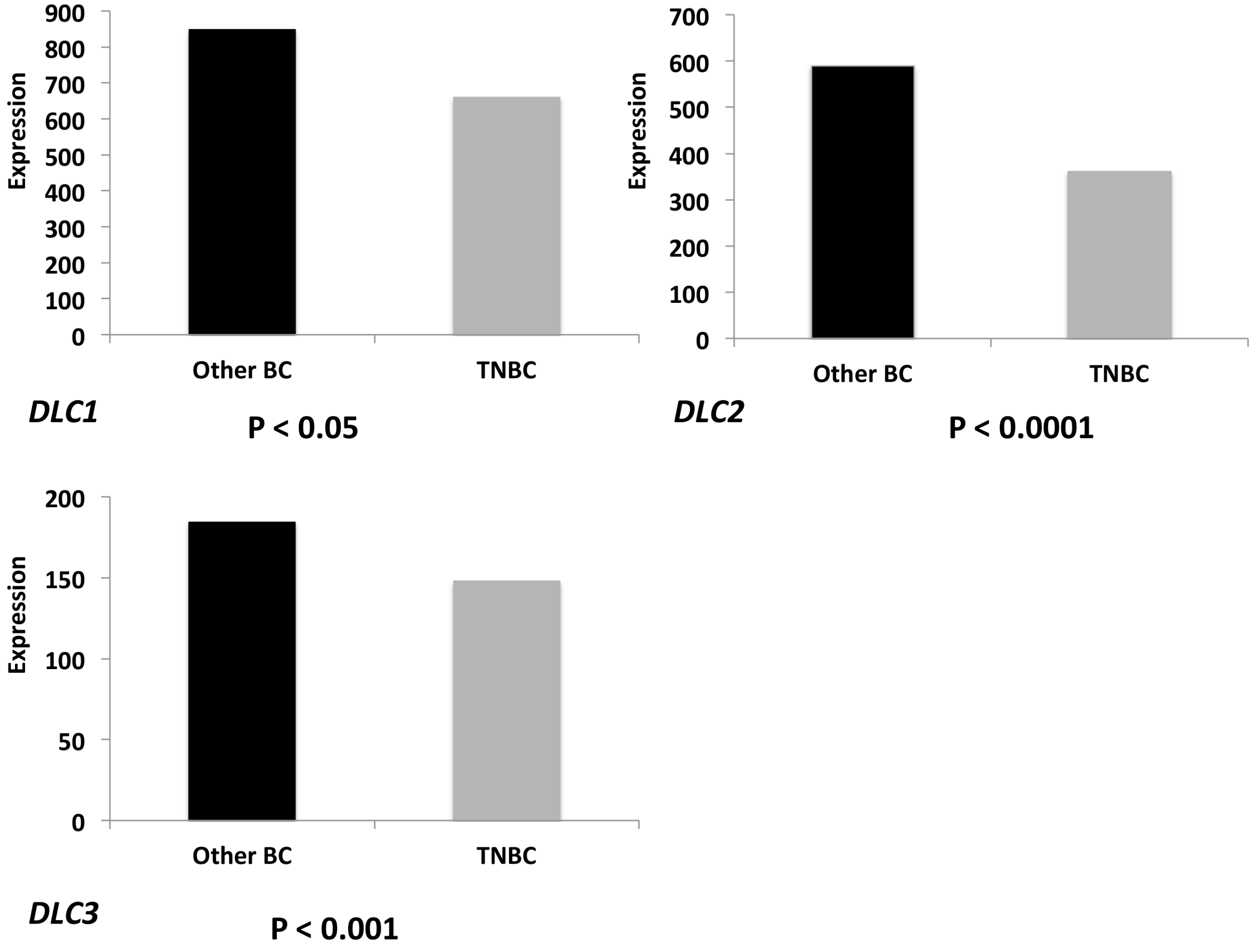

Supplementary Figure S1: *DLC* Gene expression comparison between triple-negative and non-triple-negative breast cancers. Statistical analysis was performed to compare *DLC1*, *DLC2* and *DLC3* expression in TCGA breast invasive cancer RNA-Seq dataset combined with clinical data. Medians of *DLC1*, *DLC2* and *DLC3* mRNA expression of triple-negative breast cancer (TNBC) and non-triple-negative cancer (Other BC) were calculated and the expressions were compared using Mann-Whitney U test. TNBC n = 112. Other BC n = 761.

A **DLC1 (variant 2) NM_006904 Chromosome 8: 12990095-12991174 (GRCh37)**

```
gtcgccgcgcccctcgagccagagccgcgagcccccgcccggctcaaggaggaaagtgaaccagggcttcccttcacgggttgcgaccgatccggagcccgcctggtgcgctggcccgcg
gtccccaggcaaaaggtaatcaagagtcactcctccaaaattcaaactccctccccaaactgcgagtcctgctatccccacaccacctccaagaaaatccggagactctgcagaaagcgt
ttaaagagcacagaacaggcaccgacttgacaaggcggggtgacactttctcgcggcgggtcccctccgcagcccgctcccgcggccagcccgacggcaagacgcaagtctagcttacGT
GTTAGGATCATGGTGTCCGGCTTCTTTCTGCACATCAAGCACGGCAGGCGGCGGCGGAAGCGCTGTGGGGAAGTCGAGGCAGGCGGAGGCGGCTCGGCTTCCGCGTCGGGACCCACGGCG
GCACCCGAGACGCGCGCCCTCGCGGTCCTCAACGCATCCTTGCTCGCCGCTCCCTGCCCCTCGTCACGGCCCCAGAAAGAAAGCGGGGTTTTCTAAAGATCGAAACGAGGGAGCGCTCAG
GGAGTTGGGCGAGAAGTCCGTGAGCCGGCGCTCCTGATGCGGAGAGGTGCGGCCATGTCCTGGCTGGGAGCGAAGCGCCCTCGCTCGGGCAGTCGGAGCGAACTGTCTCCCGCGCgctcc
gccagccgggccctcccgctgggcccacccccgaggggcggggccagagcgggcggcaccgcctcctccccgctgtctgggtcgcaggccttagcgacgggctgttctccggccccgcc
ccattcccaggctccgcccccgcccctcttcctcgtggccgcagcctcaggccgccctctagccccgccccgcccaggccccgcctctcctctgtcccgccccctcggttccctcctcc
cccccgccccgccccgcgcctcctccgccgaaggctggaatgtttcccccacgggcctccacgttcccagcgggaagcgccgaaaagtcgccaactattggccccgggtgcaaaggcaaa
```

B **DLC2α (variant 1) NM_178006 Chromosome 13:33860701-33858962 (GRCh37)**

```
aatgggtccttagcaggttgatgacagtatctttgaagtacctttgcaacttctgcagtgattcttgtcttaaatgattcagtgtcttaaatgtgataggcaccttctaagtattttacc
tctaggtgattaaaaattggggaagtagagaaatcaaagaggaaaggatgcttaatacgtaacgcgtgtgtactttgcctctctgagtcttatctgtaagagccctccaattcccatcac
gtgagggtcaggatctgtggtgtattcactcctggtgttaaagcccttgaagtttatttttcactggaaaagtttttatttgagttgtttcttgataatgaaggtggtggcttacgctca
gctgttgatgcttgtttgaagtagtttttttttgttgtgttgtgtttttttaaatttaagagatttatcaggtccatcttttacctggctgctgctgccctgtagaaagcgaaggagcttt
ctctaactttgacaggctgaaaagtttcctgagccctggacttgtttgtcttctacttgttttgcccttccctctctctttttctttcgactcctttagtgagggctgcttttgggtgct
tcgtcagctctgaggcacgtcaaatacgtgtattttgctttcagcgttgtgtgagggaccctggcggctggaactgcctctggtcggggcttagggctgactgcaaacgttcccattttc
ctctcttcctctcattggttcccgaggtcacgtgagggccccgggggttggaattctgagttgtggctttggcactcctcTTTCTTTTTAAAAATCGCTGGGTCTGTTGAGCTGTCCTGG
GCTGGGTGCCTTGCTCTTTGACTGAGACTGGAGACAGACGGCAACAGCCACAGGCAGACTGAGGTGGCAATAGGAAATCTGCCGAGATGTTCAGTCAGGTGCCCAGGACCCCAGCCTCAG
GCTGCTACTACCTAAATTCCATGACACCTGAGGGCCAGGAGATGTACTTGCGATTTGATCAGACTACAAGACGCTCTCCTTACAGGATGAGCCGGATTCTAGCACGCCATCAGCTAGTGA
CTAAAATTCAACAAGgtgagtggccggcagtggaaggctgttgctcattctgatttctgttggctctatttcatgctaacccagtttttttgtttgtttgtttccactttataacatat
ggatttctatgccacactacccgtaactttgaaaaataactttaggctgcagttttcagcaaacaggacagtccttagctgccacatagctcaacataaagtgcacaaaaaacttcacgg
tgggacagtgaatcataaattcccaaactgacgtgtgtctacagaacagatgagaactgttactcagtgtgtatcttaggagcttttctgcagtttcctcacactccgtcacatttaaaa
tgtggacacttgtttatttcattagggaggaggcgagggactaatgtccaccctgcccagagtatttcgaatatccttagtgaagaggaggaaagcaagaattctgttctaaaggccacc
aggctaagcactagaatcgcattctcttcctgtttgtatgtttatgtcagcagttgccacagatgtgttaatattgttttcctggtagagaattaaggtgttcgttcatctcaaaacaaa
tcccgtaacctgcacacaaaactccagcttcctaatgcaaagagaagagaatattgatta
```

C **DLC3β variant 3 NM_001142504 Chromosome X:67912911-67914230 (GRCh37)**

```
ctgccacgattaatgttcgtgccccaaattttccatgcatttctctgcggaggtagagttcagacttggggtaggggacagaagcctggggtattggggaagttgcagagccactggggc
ctgagcaacgtagggatggcagcttcctgctcaaattcctaattatctggttttcccctaccaattggagatttcactgaagttgtgtcttctggacatcccataaattatccgagcaag
tcactgaaccctcccgggcacatagcatatgtgtttaaatcccccaaacagtgccggactcattcagaactcaataaatattagcctgtgtgcctggccaagtcactgcttgagcctcag
tctttgggcttgcacctcctccctggcagcggtgtgaccggagggaaaggcagcggcgagggccttggaggacctgcaggcagtgccccgcccacccagatttgggtgactttctggcga
gtccggtgggccagactgcgcccccattggccgaggggaggtgggcggggcgtccctacagccctactcgccgacgctccctccccacttgAGCTGAGTGGACTCGGGTTGACAACGGCT
AAGCAGCCGCGGCAGCTGCTCTTCTGGCTGGCACCGTCACCCCTGCCCGACCCCAGGCCCCGCGTGTGTCCCGGACGGAGCAGTCCCTCCTGGGCTCGCCTCCTGCCCGCTCGCTCTGTG
TCAGCGCCCGAACCCAAAGGGGTGGACGCGGCCTCCAGgtaggagtctgggcgacggcagggggagatccagacaaacgggggcgcctggggctccctctccccgctttgcctctctggc
gcgctccggctcggcccttctcctagcggttccgccgggcggcgtacgcaagagacagggaggaagcgagccgagccaccccccgcccctgggattggggggtcagaatcgccgaaattgg
gggacccccgagcgcgccagccgcgcgtcttccagcgaagcctcgcgagtggggcggtggaaggcagctgtccgcctgttaccgccgggcccgtgggtctcgcagccgtcgctctggggc
tttggaggggcgttgcagctggccgggggcggcagagcttcaggctctgttgccgggtcgcgccagatctcctgccctctcctttctctgctttcgagggcatttccaggaagcccccgt
ccggctgtatgggttttcccgtcggccactgcctcattgctttctctcctgagaaatgcccaggacttcttggtattaagatagctgcggggaccaagcgaaaacgtttggacacgtctc
```

Supplementary Figure S2: DLC promoter sequences. The sequences of predominantly expressed *DLC* genes are used. **A.** *DLC1* variant 2. **B.** *DLC2* alpha, variant 1. **C.** *DLC3* beta, variant 3. The sequences of the first exons are highlighted with capital letters.

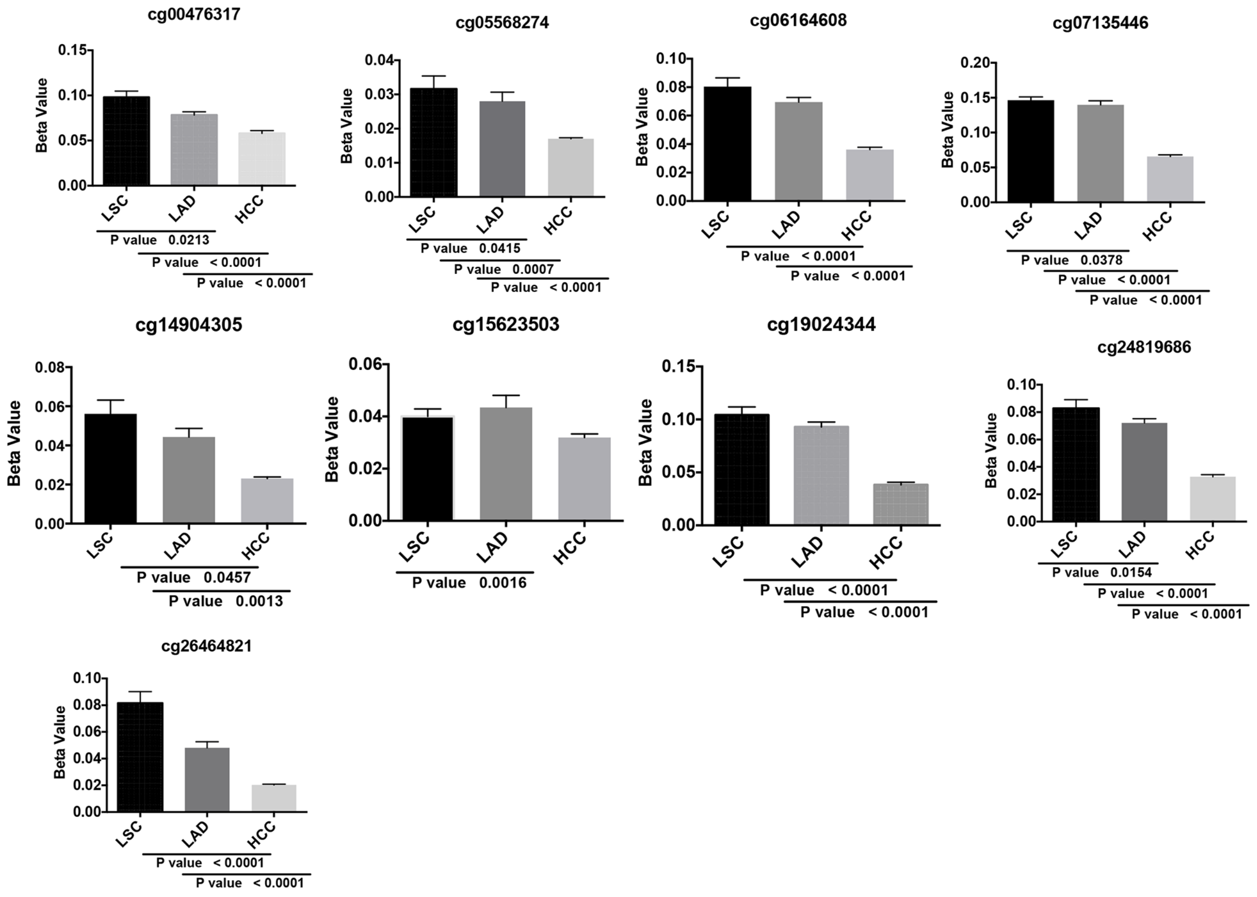

Supplementary Figure S3: *DLC1* promoter methylation in LSC, LAD and HCC. DNA methylation beta values of probes in selected regions were used for means and standard errors calculation. The result shows one of nine of *DLC1* promoter regions in LAD has higher methylation than LSC. Four of nine of *DLC1* promoter regions in LSC has higher methylation than LAD. Eight of nine of *DLC1* promoter regions in LSC and LAD have higher methylation than HCC.

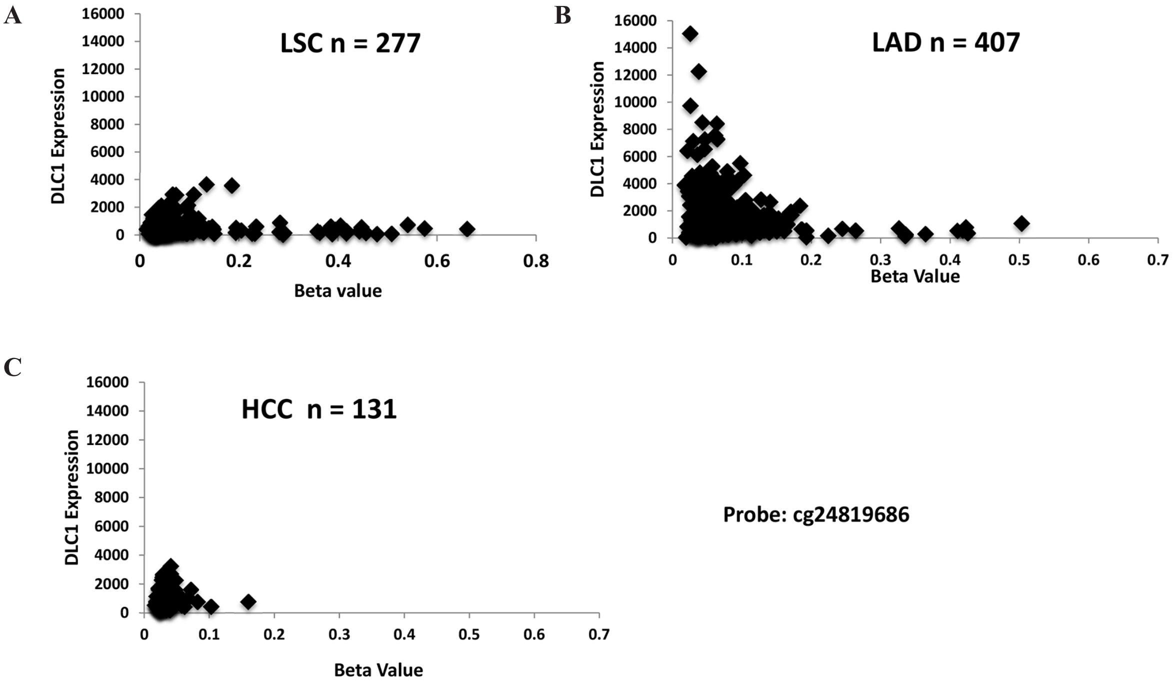

Supplementary Figure S4: Relationship between DNA methylation and *DLC1* expression in cancers. TCGA DNA methylation beta values from probe cg24819686 and *DLC1* RNA-Seq values are plotted for LSC **A.**, LAD **B.** and HCC **C.**

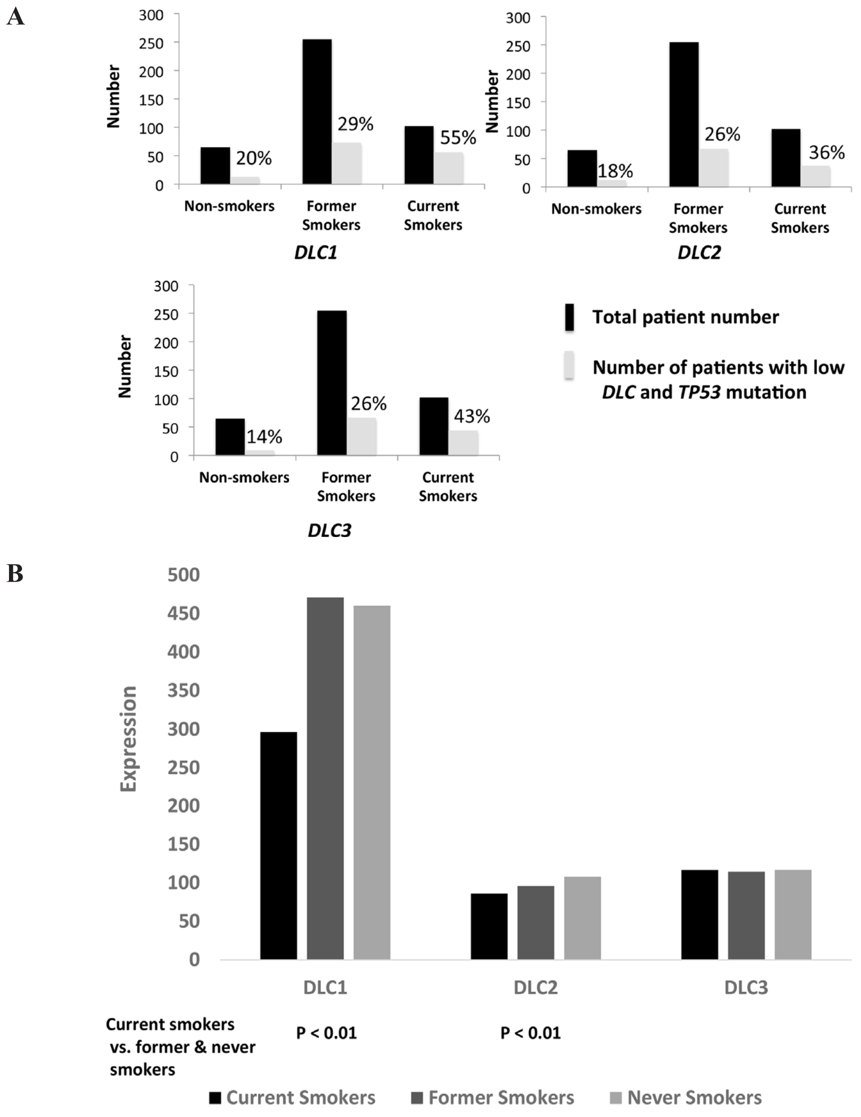

Supplementary Figure S5: Combination of *DLC1* down-regulation and *TP53* mutation is related to smoking status in lung adenocarcinoma. A. *DLC* expression and *TP53* mutations in smokers with lung adenocarcinoma from TCGA lung adenocarcinoma patients whose smoking history was known. The graph represents the number and percentage of cases in each smoker category that had both low *DLC1* mRNA expression and *TP53* mutation. **B.** Median DLC values of never smokers (n = 32), former smokers (n = 268), and current smokers (n = 49), from Director's Challenge Lung Study cohort. (lung adenocarcinoma), was calculated and compared using Mann-Whitney U test. For *DLC1* and *DLC3,* both never smokers and former smokers have higher expression than current smokers.
